# Supplementary material for: Pyruvate Dehydrogenase Kinase 1 inhibition mediated oxidative phosphorylation enhancement in cartilage promotes osteoarthritis progression
Source: BMC Musculoskelet Disord. 2023 Jul 20;24:597. doi: 10.1186/s12891-023-06585-6 (PMC10357736; doi:10.1186/s12891-023-06585-6)
Supplement: Supplementary file 1 — Additional file 1. [file 12891_2023_6585_MOESM1_ESM.zip › KEGG.Gsea.1653622432464/KEGG_LINOLEIC_ACID_METABOLISM.html]

Details for gene set KEGG\_LINOLEIC\_ACID\_METABOLISM[GSEA]

|  || Dataset | 55457\_collapsed\_to\_symbols.cls.cls #OA\_versus\_normal.cls.cls #OA\_versus\_normal\_repos |
| Phenotype | cls.cls#OA\_versus\_normal\_repos |
| Upregulated in class | normal |
| GeneSet | KEGG\_LINOLEIC\_ACID\_METABOLISM |
| Enrichment Score (ES) | -0.4004672 |
| Normalized Enrichment Score (NES) | -1.2770078 |
| Nominal p-value | 0.15905245 |
| FDR q-value | 0.29714677 |
| FWER p-Value | 1.0 |
Table: GSEA Results Summary

  

Fig 1: Enrichment plot: KEGG\_LINOLEIC\_ACID\_METABOLISM      
 Profile of the Running ES Score & Positions of GeneSet Members on the Rank Ordered List

  

| SYMBOL | TITLE | RANK IN GENE LIST | RANK METRIC SCORE | RUNNING ES | CORE ENRICHMENT || 1 | PLA2G12A | phospholipase A2 group XIIA [Source:HGNC Symbol;Acc:HGNC:18554] | 600 | 0.623 | 0.0410 | No |
| 2 | PLA2G4A | phospholipase A2 group IVA [Source:HGNC Symbol;Acc:HGNC:9035] | 2656 | 0.284 | -0.0786 | No |
| 3 | PLA2G10 | phospholipase A2 group X [Source:HGNC Symbol;Acc:HGNC:9029] | 3315 | 0.220 | -0.0988 | No |
| 4 | PLA2G3 | phospholipase A2 group III [Source:HGNC Symbol;Acc:HGNC:17934] | 3648 | 0.190 | -0.0979 | No |
| 5 | CYP3A43 | cytochrome P450 family 3 subfamily A member 43 [Source:HGNC Symbol;Acc:HGNC:17450] | 4698 | 0.096 | -0.1658 | No |
| 6 | PLA2G2D | phospholipase A2 group IID [Source:HGNC Symbol;Acc:HGNC:9033] | 5056 | 0.069 | -0.1839 | No |
| 7 | CYP2C19 | cytochrome P450 family 2 subfamily C member 19 [Source:HGNC Symbol;Acc:HGNC:2621] | 6089 | -0.011 | -0.2624 | No |
| 8 | CYP2C18 | cytochrome P450 family 2 subfamily C member 18 [Source:HGNC Symbol;Acc:HGNC:2620] | 6210 | -0.022 | -0.2687 | No |
| 9 | PLA2G5 | phospholipase A2 group V [Source:HGNC Symbol;Acc:HGNC:9038] | 6772 | -0.061 | -0.3037 | No |
| 10 | PLA2G2E | phospholipase A2 group IIE [Source:HGNC Symbol;Acc:HGNC:13414] | 7486 | -0.117 | -0.3426 | No |
| 11 | PLA2G1B | phospholipase A2 group IB [Source:HGNC Symbol;Acc:HGNC:9030] | 8232 | -0.173 | -0.3761 | Yes |
| 12 | CYP3A5 | cytochrome P450 family 3 subfamily A member 5 [Source:HGNC Symbol;Acc:HGNC:2638] | 8272 | -0.177 | -0.3542 | Yes |
| 13 | AKR1B10 | aldo-keto reductase family 1 member B10 [Source:HGNC Symbol;Acc:HGNC:382] | 8420 | -0.187 | -0.3394 | Yes |
| 14 | CYP2C9 | cytochrome P450 family 2 subfamily C member 9 [Source:HGNC Symbol;Acc:HGNC:2623] | 8595 | -0.201 | -0.3247 | Yes |
| 15 | CYP3A7 | cytochrome P450 family 3 subfamily A member 7 [Source:HGNC Symbol;Acc:HGNC:2640] | 9024 | -0.234 | -0.3250 | Yes |
| 16 | CYP2J2 | cytochrome P450 family 2 subfamily J member 2 [Source:HGNC Symbol;Acc:HGNC:2634] | 9642 | -0.287 | -0.3326 | Yes |
| 17 | PLA2G2A | phospholipase A2 group IIA [Source:HGNC Symbol;Acc:HGNC:9031] | 10152 | -0.336 | -0.3248 | Yes |
| 18 | CYP3A4 | cytochrome P450 family 3 subfamily A member 4 [Source:HGNC Symbol;Acc:HGNC:2637] | 10193 | -0.340 | -0.2801 | Yes |
| 19 | PLA2G2F | phospholipase A2 group IIF [Source:HGNC Symbol;Acc:HGNC:30040] | 10628 | -0.386 | -0.2596 | Yes |
| 20 | CYP2E1 | cytochrome P450 family 2 subfamily E member 1 [Source:HGNC Symbol;Acc:HGNC:2631] | 10988 | -0.428 | -0.2272 | Yes |
| 21 | PLA2G6 | phospholipase A2 group VI [Source:HGNC Symbol;Acc:HGNC:9039] | 11128 | -0.446 | -0.1753 | Yes |
| 22 | ALOX15 | arachidonate 15-lipoxygenase [Source:HGNC Symbol;Acc:HGNC:433] | 11405 | -0.488 | -0.1281 | Yes |
| 23 | CYP1A2 | cytochrome P450 family 1 subfamily A member 2 [Source:HGNC Symbol;Acc:HGNC:2596] | 11719 | -0.536 | -0.0770 | Yes |
| 24 | CYP2C8 | cytochrome P450 family 2 subfamily C member 8 [Source:HGNC Symbol;Acc:HGNC:2622] | 11958 | -0.585 | -0.0132 | Yes |
| 25 | JMJD7-PLA2G4B | JMJD7-PLA2G4B readthrough [Source:HGNC Symbol;Acc:HGNC:34449] | 12076 | -0.614 | 0.0640 | Yes |
Table: GSEA details [plain text format]

  

Fig 2: KEGG\_LINOLEIC\_ACID\_METABOLISM      
 Blue-Pink O' Gram in the Space of the Analyzed GeneSet

  

Fig 3: KEGG\_LINOLEIC\_ACID\_METABOLISM: Random ES distribution      
 Gene set null distribution of ES for **KEGG\_LINOLEIC\_ACID\_METABOLISM**
